# From Counting Cells to Reconstructing Death: A Commentary on Artificial Intelligence in Forensic Histopathology

**DOI:** 10.3390/diagnostics16203299

**Published:** 2026-10-11

**Authors:** Angelo Montana, Tommaso D’Anna, Francesca Licitra, Rosanna Zamparese, Loredana Buscemi, Eva Montanari, Lucia Tattoli, Maria Sofia Fede, Cataldo Raffino, Ginevra Malta

**Affiliations:** 1 Section of Legal Medicine, Department of Biomedical Sciences and Public Health, Marche Polytechnic University, 60126 Ancona, Italy; flicitra10@gmail.com (F.L.); l.buscemi@univpm.it (L.B.); evamontanari11@gmail.com (E.M.); mariasofiafede13@gmail.com (M.S.F.); 2 Department of Biomedicine and Prevention, Tor Vergata University of Rome, 00133 Rome, Italy; tommaso.danna@students.uniroma2.eu; 3 Tumor Immunology Unit, Department of Health Promotion, Mother and Child Care, Internal Medicine and Medical Specialties (PROMISE), University of Palermo, 90127 Palermo, Italy; 4 Legal Medicine Unit, University Hospital “Paolo Giaccone”, 90127 Palermo, Italy; 5 Legal Medicine Unit, Ascoli Piceno Hospital C-G. Mazzoni, Viale Degli Iris 13, 63100 Ascoli Piceno, Italy; rosannazamparese@libero.it; 6 Department of Public Health and Pediatrics, Section of Legal Medicine, University of Turin, Corso Galileo Galilei 22, 10126 Torino, Italy; luciatattoli@libero.it; 7 Legal Medicine Centre of INAIL, Via Roma 419-423, 94100 Enna, Italy; cataldo.raffino@gmail.com; 8 Department of Health Promotion, Mother and Child Care, Internal Medicine and Medical Specialties (PROMISE), University of Palermo, 90127 Palermo, Italy; ginevra.malta@unipa.it

**Keywords:** artificial intelligence, forensic histopathology, whole-slide imaging, computational pathology, postmortem interval, multimodal learning, vision-language models

## Abstract

Artificial intelligence (AI) is being investigated as a means to advance forensic histopathology from visual description to quantitative mapping, pattern recognition, and multimodal reconstruction of death. Recent developments in human postmortem analysis include validated whole-slide digitisation as foundational infrastructure and task-specific AI applications for diatom detection, glomerular quantification, coronary morphometry, myocardial classification, immunohistochemistry scoring, and virtual staining. AI-based postmortem interval (PMI) estimation remains experimental; the multi-organ study referenced in this commentary utilised an animal model, and human forensic validity has not yet been established. Foundation and vision-language models may ultimately extend these capabilities across organs and integrate microscopic findings with autopsy, toxicological, radiological, and circumstantial evidence. Postmortem tissue, however, is subject to autolysis, decomposition, agonal events, resuscitation, and non-standardised sampling, while the reference diagnosis is often a probabilistic whole-case opinion rather than an objective slide label. This work introduces FORENS-AI (FORensic Histopathology Evidence Network and Safeguards for AI), a conceptual architecture designed to convert computational outputs into traceable medicolegal evidence. FORENS-AI has not been empirically validated and is not presented as a standard, scoring system, clinical decision rule, or practice recommendation. The framework represents findings as an evidence graph, separates the Slide, Case, and Claim Gates, and proposes progressively stronger validation as outputs move from measurement to temporal, mechanistic, or causal inference. The primary aim is not to automate certification of death, but to identify safeguards to evaluate before AI-derived histological findings are permitted to inform expert opinion.

## 1. Introduction

Forensic histopathology is undergoing a transformation analogous to developments in oncological pathology, although under markedly different epistemic conditions. In surgical pathology, algorithms typically detect tumours, grade lesions, count mitoses, or predict molecular alterations. In contrast, forensic practice rarely treats the microscopic finding as the endpoint. Instead, it serves as one element within a broader reconstruction that must differentiate disease from postmortem change, establish lesion vitality, estimate the timing of lesion development, determine its contribution to death, and integrate findings with autopsy, toxicology, postmortem imaging, and contextual circumstances.

This distinction reframes the central question. The primary concern is not merely whether an algorithm can recognise a pattern, but to what extent that pattern supports a medicolegal proposition. For example, a model may accurately quantify 80% coronary stenosis without demonstrating fatal ischemia; detect diatoms without establishing drowning; classify myocardial necrosis without determining its temporal relationship to death; or identify pulmonary oedema without distinguishing among cardiac failure, intoxication, resuscitation, and agonal change. In forensic histopathology, analytical correctness and evidentiary relevance are related yet fundamentally distinct concepts.

Digital whole-slide imaging, which underpins histological AI, has already demonstrated practical utility in forensic contexts. Whole-slide images (WSIs) can be converted into spatially indexed datasets, enabling computational detection, measurement, and comparison of cells and tissue structures. In the multicenter iForensic validation study, six forensic pathologists evaluated 100 slides—comprising 80 hematoxylin and eosin (H&E) and 20 special-stain slides—and achieved a 97.8% mean concordance between digital and conventional microscopy [1]. This finding establishes an infrastructural foundation, rather than direct AI validation, indicating that forensic histology can be digitised with sufficient fidelity to enable subsequent computational analysis.

The discipline is advancing from isolated measurements toward foundation models, vision-language systems, and multi-organ representations. This evolution could be transformative. Rather than examining a limited number of fields, pathologists could receive comprehensive maps of relevant structures, prioritised regions for review, quantitative comparisons across organs, alerts regarding autolysis or staining artefacts, and a structured synthesis linked to each supporting slide coordinate. The appropriate goal is not the creation of an autonomous ‘cause-of-death algorithm’, but rather a forensic histology copilot that enhances the observable and measurable aspects of a case while maintaining clear distinctions among microscopic observation, diagnostic interpretation, and causal opinion.

This Commentary reviews both established and emerging applications of AI in forensic histopathology, addresses the limitations of conventional computational pathology paradigms for postmortem material, and identifies errors that pose particular risks in medicolegal inference. It introduces FORENS-AI as a conceptual framework for linking algorithmic performance to the evidentiary scope of resulting conclusions. This Commentary does not present an original dataset, model-training experiment, or validated scoring system. The gates, workflow, and claim levels discussed are intended as qualitative safeguards and research propositions, rather than operational thresholds, clinical guidelines, standards of practice, or recommendations for current casework.

Existing responsible-AI frameworks in forensic science, such as the Responsible Artificial Intelligence Framework described by Stacey and colleagues [2], address the organisational lifecycle of AI projects through governance principles, documentation, approval processes, and project registration. In contrast, FORENS-AI focuses on the more specific issue of the evidentiary reach of AI-derived histological findings within individual postmortem cases. Its unique contribution lies in connecting slide provenance and analytical adequacy with decedent-level integration, and in constraining output language to a cumulatively validated claim level. FORENS-AI is therefore designed to complement, rather than replace, organisational governance frameworks.

Recent scoping and systematic reviews reveal a heterogeneous forensic-AI evidence base, largely composed of task-specific studies, and highlight ongoing challenges related to generalisation, interpretability, and validation [3,4]. This body of literature underscores the necessity for domain-specific safeguards but does not define the components or thresholds of FORENS-AI. The studies referenced in this Commentary were chosen to illustrate the current evidence base, relevant capabilities, and principal limitations; they were not identified through systematic review methods and do not represent a comprehensive synthesis of the literature.

## 2. From Detection to Quantitative Postmortem Phenotyping

The most mature application of AI is converting microscopic structures into reproducible measurements. Researchers have used convolutional networks to detect diatoms in forensic WSIs [5]. A kidney pipeline analysed 67,621 glomeruli from 86 autopsies, corresponding to 786 ± 277 glomeruli per individual [6]. A transformer-based system segmented the coronary lumen and internal and external elastic lamina, calculating stenosis with a mean absolute error of 1.01 percentage points and an intraclass correlation coefficient of 0.995 [7]. The latter result was obtained on only seven test regions, illustrating both the precision achievable and the limited evidentiary strength of small, selected validation sets. Such systems can produce more than counts. For object class, the estimated density is

$${\hat{D}}_{k}=\frac{{\hat{N}}_{k}}{A_{\mathrm{valid}}},$$

Density is defined as the number of objects per square millimetre, where N_k_ is the algorithm-estimated object count, and A_valid_ is the analyzable tissue area after excluding regions affected by folds, blur, tissue loss, or severe autolysis. Future research may report density estimates with case-level uncertainty intervals when these can be reliably determined. Algorithms can also quantify size distributions, staining intensities, distances from vessels, clustering indices, and inter-organ variability. Comparable segmentation pipelines can quantify glomerulosclerosis, myocardial fibrosis, hepatic steatosis, inflammatory infiltrates, haemorrhage, microthrombi, fat or bone-marrow emboli, vascular remodelling, and immunoreactive areas.

This development marks a transition from selective visual sampling to computational phenotyping. Whereas a pathologist typically examines several high-power fields, algorithms can analyse entire tissue sections, identify the most information-rich regions, and maintain a coordinate-linked audit trail. These capabilities support informed block selection for deeper sections, special stains, immunohistochemistry, or molecular analysis. For example, in suspected myocarditis, a model can map inflammatory foci, assess their relationship to myocyte injury, and identify fields requiring immunophenotyping. In suspected embolism, algorithms can systematically search multiple lung sections, thereby reducing reliance on incidental visual findings. Artificial intelligence has also been utilised to classify forensic morphology. In a proof-of-concept study with 150 myocardial images (50 normal, 50 acute infarction, and 50 old infarction), the InceptionResNet-v2 model achieved an accuracy exceeding 95% [8]. Generative vision AI subsequently evaluated 225 forensic immunohistochemical images across five immunoreactivity categories. Blind testing on 75 images resulted in an overall accuracy of 81.3% and a quadratically weighted κ of 0.848. Binary sensitivity and specificity reached 98.3% and 93.3%, respectively, while intermediate staining categories exhibited errors up to 33.3% [9]. This distinction matters in forensic contexts. Clear positive results are reliably identified, whereas borderline patterns, which depend on cell identity, distribution, and case context, remain less stable.

Objective image-based assessment of postmortem histology predates current deep-learning systems. In human cartilage, semi-automatic colour deconvolution and mean grey-value analysis demonstrated strong correlation with visual Bern scores across five stains (r ≥ 0.77), and excellent inter-rater reliability (ICC ≥ 0.965) [10]. This work demonstrates that transparent, auditable quantification can reduce observer dependence, although it does not independently validate continuous postmortem interval estimation.

Image-to-image models offer further opportunities. Researchers have developed virtual hematoxylin and eosin (H&E) staining using more than 16,000 paired images of autopsy tissue, totalling over 0.7 terabytes of data [11]. These models may enable recovery of interpretable morphology from label-free tissue, facilitate rapid screening, or standardise staining appearance. However, a virtual stain is a generated representation. If an algorithm reconstructs a nucleus or cytoplasmic boundary not directly observable in the original acquisition, the generated feature does not inherently possess the evidentiary status of a conventional histochemical observation. Table 1 summarises these applications and their evidentiary limitations.

External-centre validation refers to evaluating data from an institution not involved in model development. In contrast, a held-out test set from the same institution does not constitute external-centre validation.

## 3. The Emerging Multi-Organ Forensic Histology Copilot

A potential research architecture for future forensic artificial intelligence may integrate multiple interacting modules rather than relying on a single classifier. Initially, a postmortem quality-control module could identify issues such as blur, folds, staining failure, tissue loss, autolysis, putrefaction, and microbial overgrowth. Organ-specific encoders could then represent cells, structures, and lesions, while task-specific components might perform segmentation, morphometry, immunohistochemical scoring, pattern classification, or experimental temporal estimation. A case-level aggregator could compare findings across organs and tissue blocks. Finally, a multimodal layer could relate histological data to gross findings, postmortem radiology, toxicology, microbiology, biochemistry, clinical history, and circumstances. These components are proposed for evaluation and do not represent specifications for a currently deployable system.

Foundation models trained on extensive whole slide image (WSI) collections can provide transferable tissue representations and may reduce the number of annotated forensic slides required for specific tasks. These models could facilitate few-shot detection of rare findings, similarity-based retrieval of previous cases, interactive segmentation, and cross-organ comparisons. However, most clinical foundation models have been trained on material from living patients, standardised resection specimens, and disease-centred datasets. Their performance cannot be presumed to transfer directly; any forensic application would necessitate explicit evaluation and adaptation to postmortem material.

Experience in clinical computational pathology demonstrates that high internal discrimination does not guarantee transportability or safe application. External and temporal validation across institutions and workflows, explicit calibration, uncertainty estimation, out-of-distribution (OOD) testing, and abstention policies are essential for successful translation [14,15,16,17,18]. Reader studies indicate that validated artificial intelligence assistance can enhance pathologist performance while maintaining expert adjudication [19]. The FORENS-AI framework incorporates these principles as research propositions and introduces a forensic-specific question: whether uncertainty could determine both the availability of an output and the medicolegal scope of the proposition it supports.

Vision-language models may enable pathologists to query whole slide images using natural language, retrieve regions consistent with specified patterns, and generate draft descriptions. The SongCi model is a strong proof of feasibility. It integrated WSIs from nine organs with gross-finding text and diagnostic queries, utilising more than 16 million patches, 2228 image-language pairs, 228 decedents, and 471 diagnostic outcomes [13]. Mean recall decreased from 0.823 internally to 0.778 and 0.770 in two external cohorts, while mean intersection over union declined from 0.682 to 0.617 and 0.594. This external decline indicates that multimodal forensic representations are feasible but not yet fully generalizable across institutions.

We propose an evidence graph, rather than an unrestricted report, as a candidate operational output for future evaluation. Each node would represent a finding, including its organ, slide, coordinates, quantitative value, uncertainty, quality indicators, and acquisition method. Edges could indicate whether one finding supports, contradicts, qualifies, or is conditionally dependent on another. For instance, in a suspected cardiac death, the graph could connect coronary stenosis, myocardial fibrosis, acute ischemic changes, heart weight, postmortem coronary imaging, and toxicology. The system could retrieve suspicious regions, quantify chronic substrates, and flag discordant findings, while a claim-limiting rule would prevent severe coronary atherosclerosis alone from being reported as fatal.

A retrieval-constrained language model, followed by deterministic claim-level validation, could generate prose exclusively from verified graph nodes. Each sentence would remain linked to the corresponding slide region or ancillary result. Unsupported additions, unverified temporal statements, and causal language exceeding the validated claim level would be flagged or withheld by a rule-based claim checker. Such a system would modify the pathologist’s work not by replacing microscopic interpretation but by separating exhaustive machine observation from expert contextual adjudication.

## 4. Why Postmortem Computational Pathology Is Different

Postmortem histology reflects two superimposed and interacting processes: the disease or injury under investigation and the tissue changes that occur during the dying process and after death. Agonal hypoxia, circulatory failure, resuscitation, autolysis, bacterial decomposition, refrigeration, fixation delay and variable formalin penetration alter morphology and antigenicity in organ-specific ways. Autolysis can resemble necrosis; contraction bands may reflect ischemia, catecholamine release or resuscitation; edema and congestion may be genuine but nonspecific; bacterial growth may represent decomposition rather than infection.

These transformations create a three-domain problem. The observed image ***X*** may be considered a function of the target biological lesion ***L***, postmortem transformation ***P*** and technical acquisition ***T***:

***X*** = ***f***(***L***,***P***,***T***) + ***ε***.

Here, ***f*** may include interactions among the target lesion, postmortem transformation and technical acquisition. ***P*** and ***T*** may act as nuisance domains when the target is *L*; conversely, *P* may itself become the target in experimental applications such as postmortem interval (PMI) estimation.

These generic representations make sources of biological and technical variation explicit; they are not scoring rules, weights or operational thresholds proposed for FORENS-AI.

A conventional model trained to predict ***L*** from ***X*** may instead exploit ***P*** or ***T***. If cases of one diagnosis were scanned at one institution, fixed for a different duration or associated with a longer PMI, excellent test performance may reflect an institutional or postmortem signature rather than lesion recognition.

The reference standard is equally problematic. Forensic histology frequently confirms, excludes or qualifies a hypothesis generated by the autopsy rather than independently determining the cause of death [20]. Labels such as “acute myocardial infarction”, “vital injury” or “drowning” may have been assigned after the expert reviewed the same histology, together with the circumstances and ancillary findings subsequently supplied to the algorithm. Training on such labels risks circularity. PMI and wound age references are often intervals rather than exact times, and causal contribution is an evaluative conclusion conditioned by competing explanations [21].

Forensic claims may therefore be modeled propositionally. If ***H*****_1_** denotes the hypothesis that a finding supports a proposed mechanism and ***H*****_0_** a relevant alternative, a potentially useful quantity is not merely a class probability but the likelihood ratio based on the observed AI-derived output:

(1) $$LR_{\mathrm{AI}}\left(e\right)=\frac{f_{E|H_{1}}\left(e\right)}{f_{E|H_{0}}\left(e\right)},$$

where ***e*** is the observed AI-derived score and $f_{E|H_{j}}\left(e\right)$ denotes its conditional density under hypothesis ***H_j_***; for discrete outputs, conditional probabilities replace densities.

Estimating such ratios would require representative alternative hypotheses and independently adjudicated cases. Until these data exist, model probabilities cannot be interpreted as evidentiary likelihood ratios or posterior probabilities of cause of death.

## 5. Forensic-Specific Algorithmic Errors

A common forensic failure involves learning a genuine but irrelevant signal. Neural networks may identify fixation, scanner type, staining batch, sampling convention, decomposition severity, or reporting style rather than actual pathology. In clinical histology, maintaining acquisition-site integrity during cross-validation reduced the area under the receiver operating characteristic curve by a mean of 0.069 across 58 tasks, with performance deterioration observed in 51 of 56 initially predictable targets [22]. PathoROB detected robustness deficits in all 20 evaluated pathology foundation models and demonstrated that the centre of origin could be predicted from their embeddings with 88–98% mean accuracy [23]. The heterogeneity of postmortem material likely exacerbates these shortcut-learning behaviours.

Data leakage may arise when tiles, slides, or organs from the same decedent are distributed across different data partitions. The appropriate unit of independence is the decedent rather than the image patch. Models may also internalise the pathologist’s selection behaviour; for example, a region preselected as “representative infarction” is not equivalent to an unselected myocardial whole slide image. Consequently, high image-level accuracy may reflect classification within curated regions rather than detection performance in routine clinical practice.

Additional errors include unwarranted certainty in decomposed tissue, the disappearance of rare lethal entities within majority classes, the use of incompletely investigated cases as negative controls, and the hallucination of plausible morphology in generated descriptions. The most significant error is claim escalation, in which an object detection is interpreted as a diagnosis, a diagnosis is inferred as a mechanism, and a mechanism is ultimately attributed as the cause of death without independent supporting evidence.

AI assistance can influence expert reasoning even when algorithmic outputs are intended to be advisory. A fluent narrative or seemingly high numerical confidence may anchor initial interpretations, promote automation bias—the acceptance of erroneous recommendations—and direct subsequent review toward concordant features, thereby reinforcing confirmation bias. These issues are pertinent to the field: irrelevant contextual information has been experimentally shown to affect forensic pathologists’ manner-of-death determinations [24]; both pathologists and trainees have demonstrated a willingness to rely on AI advice in experimental grading tasks [25]; and agreement with incorrect AI recommendations has been documented in separate diagnostic decision-support experiments [26]. Neither apparent confidence nor explanatory fluency ensures proper calibration, especially under postmortem domain shift. FORENS-AI proposes several process safeguards for future evaluation: the pathologist should record the initial forensic question and relevant alternatives prior to viewing AI output; native slides and source data should remain independently accessible; supporting findings, contradictions, and missing evidence should be displayed separately; and each output should specify its validated claim level and any reason for abstention. High-impact or discordant outputs may undergo independent secondary review. Future reader studies should assess not only accuracy but also changes in opinion following AI exposure, erroneous acceptance of AI advice, expert overrides, and unsupported claim escalation. These safeguards are intended as hypotheses for empirical validation rather than as evidence that cognitive bias has been eliminated.

## 6. FORENS-AI: A Proposed Claim-Calibrated Conceptual Framework

FORENS-AI (Forensic Histopathology Evidence Network and Safeguards for AI) is introduced as a conceptual architecture for studying and managing claim escalation. It does not constitute a validated standard, certification scheme, scoring system, clinically deployable product, or practice recommendation. The following components identify candidate variables for measurement in future studies, without prescribing a universal mathematical combination or threshold. Any operational rule would require task-specific derivation, independent calibration, external validation, and professional consensus.

FORENS-AI organises case information as a directed, typed evidence graph. Each observation node records the finding, organ, slide and spatial coordinates, originating raw data, quality indicators, estimated uncertainty, and computational provenance, including model version and processing history. Auditable edges indicate whether observations support, contradict, qualify, or depend on one another. This structure constitutes a provenance-and-reasoning graph rather than a causal directed acyclic graph. Provenance links are deterministic, while inferential links may be rule-based or expert-curated, explicitly versioned, and open to review. The objective is to ensure that every case-level statement is traceable to the specific slide region, ancillary result, and computational operation from which it originated.

FORENS-AI delineates three sequential conceptual gates for future evaluation.

1. Slide Gate. The Slide Gate evaluates whether an image is suitable for a specified analytical task. Relevant dimensions include focus, tissue integrity and completeness, stain adequacy, autolysis, native-image availability, exclusion maps, independently validated task performance, and uncertainty estimates. These dimensions are maintained separately; they are not combined into a universal adequacy score, assigned weights, or permitted to compensate for a task-critical failure. In a validated implementation, a critical failure would prompt abstention even if other dimensions are acceptable. For example, a slide may be adequate for glomerular counting but inadequate for subtle nuclear classification. Any generated or virtually stained image would remain paired with its source image and be clearly identified as derivative.

For future evaluation, FORENS-AI conceptualises uncertainty as a candidate referral policy rather than solely as a numerical score. For each task, an operating threshold and the associated risk-coverage trade-off would be prespecified using independent calibration data. A validated system would withhold task-specific outputs and refer cases for direct expert review when a critical quality dimension fails, the case falls outside the demonstrated domain, prediction sets are excessively broad, independently trained models show substantial disagreement, or the requested claim relies on missing evidence modalities. Referral would direct the pathologist to the native slide and source data, rather than to an AI-generated explanation. Since uncertainty estimates may fail under domain shift, they are intended to complement, not replace, external validation and human review [15,16].

2. Case Gate. The Case Gate evaluates whether slide-level outputs remain valid at the decedent level. In future validation studies, all slides, blocks, and organs from a single individual would be retained within the same data partition, and generalisation would be assessed across centres, scanners, stains, fixation intervals, postmortem intervals, decomposition states, and sampling practices. Correlated observations, such as multiple tiles from a single microscopic focus, would be grouped into substantially independent evidence clusters prior to case-level synthesis. Supporting findings, contradictions, and missing evidence would be presented separately. Unless an aggregation rule has been empirically derived and calibrated, no weighted sum of these elements should be interpreted as a probability, likelihood ratio, or validated evidentiary score. Insufficient effective evidence or unresolved contradictions would prompt abstention from automated synthesis.

The Case Gate also designates domain shift as a candidate target for validation. Potential methods include distance-based, density-based, disagreement-based, and conformal prediction approaches; however, no single method can be presumed effective across all tasks or centres. Any threshold would be derived using data not utilised for model fitting and would be tested in genuinely external cohorts [14,17]. Cases exhibiting unusual decomposition, rare pathology, previously unseen scanners, or atypical organ combinations would be flagged as outside the demonstrated domain and referred for direct expert review.

3. Claim Gate. The Claim Gate is intended to restrict the language of any future validated output to the highest empirically supported level. Level 1 encompasses detection and measurement, such as “37 diatom-like objects were detected” or “estimated luminal stenosis is 78%.” Level 2 involves tissue-level classification, for example, “the myocardial pattern is compatible with established fibrosis.” Level 3 includes temporal or mechanistic support only when the specific model, population, and intended claim have undergone independent validation. For instance, the animal PMI evidence summarised in Table 1 does not support a Level 3 human PMI claim. Level 4 involves organising multimodal evidence, detecting contradictions, and ranking hypotheses relevant to determining the cause or manner of death.

Validation is conceptualised as cumulative and non-transitive: success at Level 1 does not authorise Level 2, nor does success at Level 2 authorise Level 3 or Level 4. Advancement to a higher claim level requires successful independent validation at all lower levels, availability of the necessary modalities for the proposed statement, acceptable case quality and uncertainty, and evidence that the case remains within the validated domain. This Commentary does not propose universal numerical cutoffs; future validation should test progressively more restrictive operating conditions as the medicolegal scope of a claim expands.

Level 4 does not authorize autLevel 4 does not permit autonomous certification of the cause or manner of death. The final opinion remains the responsibility of an expert, as it requires evaluation of alternative propositions, normative judgment, and information not reducible to histological data. Any future FORENS-AI output would specify its highest permitted claim level, source regions, model and dataset versions, quality and uncertainty indicators, contradictions, missing evidence, and the reason for any abstention. The empirical hypothesis for future testing is that claim-constrained systems will generate fewer unsupported causal statements than otherwise comparable unconstrained systems, without significantly reducing detection of relevant findings; this Commentary does not test that hypothesis.

## 7. Proposed Integration into a Forensic Histopathology Workflow

To illustrate potential interactions between the framework and forensic practice, Table 2 presents a hypothetical case workflow for future evaluation, not a current practice recommendation. Expert judgment anchors the process at both the initial and final stages. Artificial intelligence would organise, quantify, and maintain the traceability of observations, while the forensic pathologist would interpret their evidentiary significance within the context of the entire case.

For instance, in cases of suspected cardiac death, a future implementation may employ the Slide Gate to identify poorly preserved myocardial regions and to quantify coronary luminal narrowing or established fibrosis. The Case Gate may then compare these findings with gross coronary observations, heart weight, postmortem imaging, toxicology results, and resuscitation history. Subsequently, the Claim Gate could prevent measured stenosis or fibrosis from being mischaracterised as fatal acute ischemia. If temporal or mechanistic inference has not been externally validated in comparable human postmortem material, the output would remain a measurement or tissue-level classification, and the report would retain unresolved alternatives. This scenario shows the framework’s intended function rather than a currently validated capability.

## 8. Priorities for Empirical Validation

**Figure 1:**
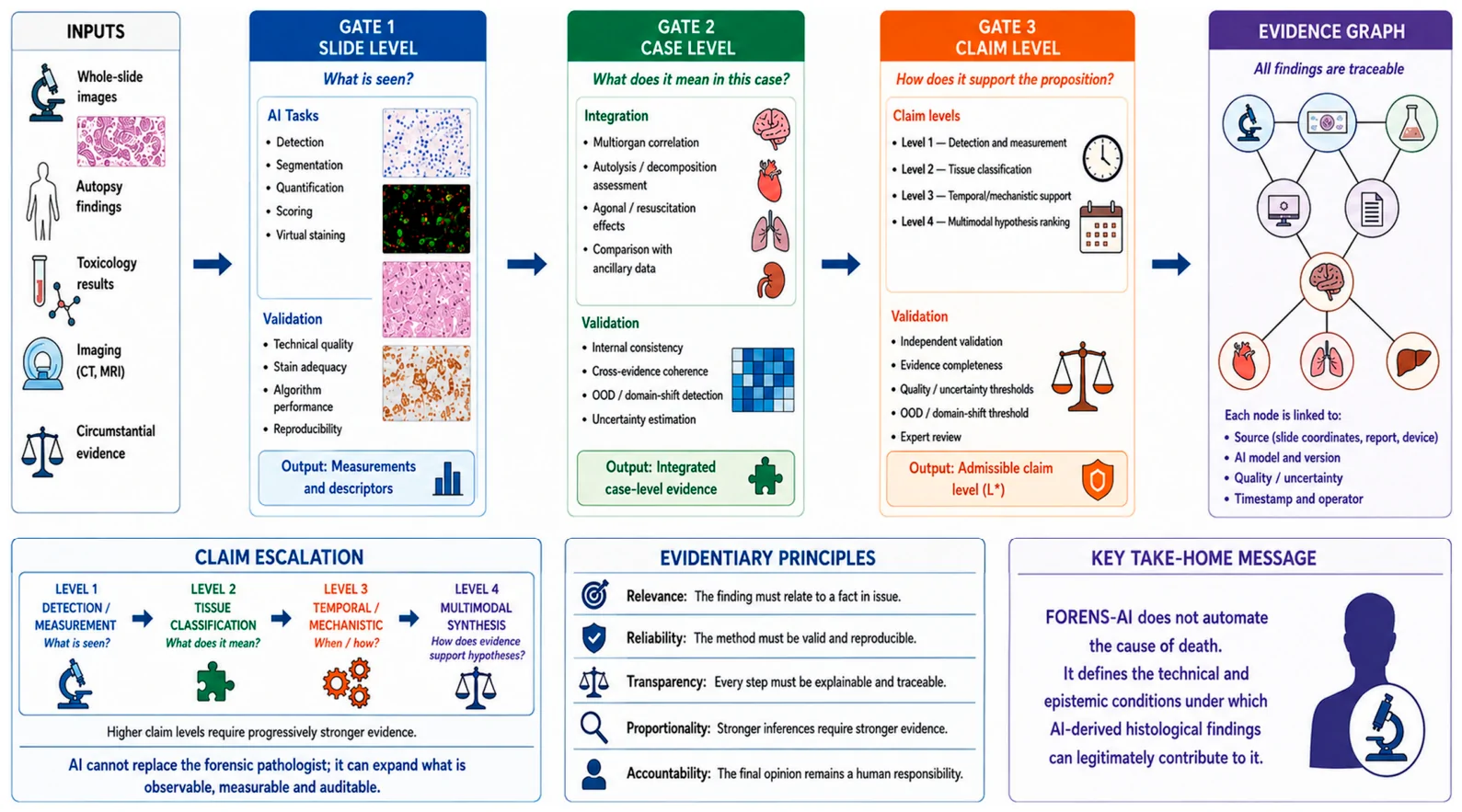
Conceptual overview of the proposed FORENS-AI framework for AI-assisted forensic histopathology. In future evaluation, a provenance-aware evidence graph would be examined together with three sequential conceptual gates: the Slide Gate would evaluate task-specific image adequacy and analytical validity; the Case Gate would assess decedent-level coherence, uncertainty and domain shift; and the Claim Gate would restrict outputs to the highest cumulatively validated claim level. The claim levels range from detection and measurement to tissue classification, temporal or mechanistic support and, at Level 4, multimodal evidence organization and hypothesis ranking. None of these functions has been validated as an integrated workflow, and Level 4 does not authorize autonomous certification of the cause or manner of death, which remains an expert forensic determination.

A future empirical program should evaluate three distinct levels (Figure 1). Analytical validation would assess task-specific detection, segmentation, discrimination, and calibration. Case-level validation would test generalisation across decedents, centres, and postmortem conditions. Forensic utility studies would determine whether the system improves region selection, use of ancillary tests, reporting time, and interobserver agreement, while maintaining appropriate abstention and preventing escalation of unsupported claims.

Each future study should be task-specific. All material from a single decedent would remain within one data partition, and final evaluation would utilise an independent centre or a later temporal cohort whenever feasible [17]. Sample size determination would consider target prevalence, primary endpoint, desired precision, clustering by decedent and centre, expected exclusions, and planned subgroup analyses; no universal minimum can be specified at this conceptual stage [27]. Performance measures would vary by task. For example, experimental temporal estimation would be evaluated using absolute error and interval coverage rather than broad time-bin accuracy alone, while calibration and risk-coverage behaviour would accompany discrimination for models capable of abstention.

A focused proof-of-concept study could compare claim-constrained and unconstrained outputs on the same cases, followed by blinded adjudication by forensic pathologists. A candidate outcome is the unsupported claim escalation rate, defined as the proportion of AI-generated statements that exceed the highest claim level supported by the prespecified reference evidence. This candidate measure requires independent reproducibility and validity assessment, and would be reported alongside sensitivity for relevant findings, diagnostic performance where applicable, abstention frequency, risk-coverage behaviour, and reporting time. Reader studies could obtain unaided opinions before AI exposure and subsequently measure opinion changes, erroneous acceptance of AI advice, expert overrides, and anchoring on the first displayed output. These experiments are proposed as future tests of FORENS-AI; no such result is reported in the present Commentary.

Following publication of this Commentary, the authors intend to develop and initiate a multicenter validation study of FORENS-AI. The proposed protocol would begin with a limited number of prespecified forensic histopathology tasks and incorporate harmonised decedent-level data structures, blinded expert adjudication, reader-study measures of cognitive bias, claim-escalation endpoints, and external-centre testing. Protocol development, participating-centre agreements, ethics and data-governance approvals, funding, and prospective registration would be addressed prior to recruitment or analysis. This statement describes a future research intention and does not imply that a protocol, consortium, or empirical validation is already in place.

Governance would develop in parallel and remain proportionate to the intended use and the highest permitted claim level. Organisational responsible-AI frameworks [2] and the evidentiary safeguards proposed here address complementary aspects of this process. Only prospective and externally validated evidence could justify subsequent professional consensus, audit criteria, or standardisation. Until such evidence is available, FORENS-AI remains a proposed research architecture.

## 9. Conclusions

Artificial intelligence is advancing forensic histopathology from selective visual examination toward quantitative, searchable, and potentially multimodal evidence analysis. In human postmortem material, the most developed applications include digitisation, detection, morphometry, and selected classification tasks. Temporal estimation remains experimental, and the multi-organ evidence discussed here comes from animal models rather than validated human casework. Vision-language and multimodal systems are also still emerging. The primary challenge is not only whether an algorithm can identify microscopic patterns, but whether the evidentiary strength of those patterns is sufficient to justify the medicolegal statements generated from them.

FORENS-AI presents a conceptually explicit and testable research proposition. By representing findings in an evidence graph, applying the Slide, Case, and Claim Gates, documenting uncertainty and contradiction, and distinguishing four levels of inferential reach, the framework aims to maintain the distinction between quantifying structures and reconstructing cause of death. The value of FORENS-AI will depend on whether external and multicenter validation demonstrates that claim-calibrated systems improve forensic practice and reduce erroneous causal attribution. The authors plan to develop a multicenter validation study to evaluate the framework on prespecified forensic histopathology tasks following publication of this Commentary. Until such validation is completed, FORENS-AI should be regarded as a research proposal rather than practice guidance. While AI may increase the visibility, measurability, and traceability of microscopic evidence, it cannot render the final opinion more certain than the underlying evidence allows.

## Figures and Tables

**Table 1:** Present and emerging AI functions in forensic histopathology.

AI Function	Reported Evidence, Study Material and Validation	Potential Forensic Use	Evidentiary Limitation
Detection and counting	Automated diatom detection in human tissue digests with independent casework test samples from five drowning cases, but no external-center validation [5]; 67,621 glomeruli analyzed in 86 human autopsies in a single-center pilot cohort without independent external validation [6]	Exhaustive screening, density estimation and spatial mapping	Presence or count alone does not establish drowning, disease severity or causal relevance
Segmentation and morphometry	Coronary stenosis MAE 1.01 percentage points and ICC 0.995 in human judicial-autopsy tissue; internal 82/14/7 region-of-interest split and no external-center validation [7]	Quantification of lumen, vascular walls, fibrosis, steatosis, hemorrhage and embolic material	Morphometric severity is not equivalent to mechanism or cause of death
Morphological and IHC classification	Myocardial classification above 95% in 150 human images with internal testing [8]; IHC accuracy 81.3% in 75 held-out human forensic images from the same institution, with no external-center validation [9]	Triage, second reading, injury-pattern recognition and standardized scoring	An image class does not establish lesion age, vitality or causal contribution
Image reconstruction	Virtual staining trained on human lung-autopsy tissue from eight cadavers and blind tested on ten unseen decedents; no external-center validation [11]	Rapid visualization and recovery of morphology from label-free tissue	Generated features lack independent evidentiary status unless verified against native or conventionally stained material
Temporal and multi-organ inference	Four-class PMI accuracy of 93.75% internally and 87.5% in an author-designated external set comprising four Bama minipigs; no human validation [12]	Quantification of coordinated postmortem change	Animal time-bin classification is not equivalent to continuous human PMI estimation
Vision-language analysis	More than 16 million patches and 2228 WSI-language pairs from human postmortem material; evaluated in two independent external institutional cohorts [13]	Cross-organ retrieval, diagnostic ranking and structured description	Generated language does not itself establish the cause or manner of death and cannot constitute autonomous certification.

Abbreviations: AI, artificial intelligence; ICC, intraclass correlation coefficient; IHC, immunohistochemistry; MAE, mean absolute error; PMI, postmortem interval; WSI, whole-slide image; κ, quadratically weighted kappa coefficient.

**Table 2:** Illustrative integration of FORENS-AI into a future forensic histopathology workflow. This sequence is proposed for empirical evaluation and is not a validated practice standard.

Workflow Stage	Proposed AI-Supported Function	Forensic-Pathologist Checkpoint	Output Boundary
Case framing	Register the case question, specimen map, intended analytical task and permitted contextual inputs; create an audit trail.	Record the provisional question and relevant alternative hypotheses before viewing AI output; confirm sampling and task suitability.	Task specification only; no diagnostic or causal conclusion.
Slide Gate	Map tissue coverage, focus, staining quality, autolysis, artifacts and possible out-of-domain features, with every flag linked to native-slide coordinates.	Inspect the native slide and excluded regions; decide whether analysis can proceed or requires recutting, restaining or direct manual review.	Task-specific fitness statement, with abstention when a critical dimension fails.
Analytical review and Case Gate	Return source-linked detections, measurements or classifications and display supporting findings, contradictions and missing evidence alongside ancillary case information.	Confirm or reject flagged regions, assess the independence of observations and compare them with gross autopsy findings, postmortem imaging, toxicology and other relevant investigations.	Levels 1–2 unless a higher claim has been separately and externally validated for the relevant domain.
Claim Gate	Generate draft language restricted to the highest validated claim level and disclose uncertainty, domain limitations, model version and reasons for abstention.	Evaluate alternative propositions, request independent review for high-impact or discordant outputs where appropriate, and edit or override the draft.	No temporal, mechanistic, cause-of-death or manner-of-death statement beyond the demonstrated validation level; no autonomous certification.
Reporting and audit	Preserve slide coordinates, source data, model and dataset versions, abstentions, user edits and overrides.	Author and sign the final opinion, document material disagreements with the AI output and review errors or near misses.	The medicolegal opinion remains attributable to the forensic expert.

## Data Availability

No new data were created or analyzed in this Commentary. Data sharing is not applicable.
